# CPAK classification shows limited side‐to‐side agreement and limited reliability as a standalone surrogate for constitutional alignment in rheumatoid arthritis knees

**DOI:** 10.1002/jeo2.70942

**Published:** 2026-10-06

**Authors:** Toshiyuki Omori, Tomohiro Kayama, Teruyuki Miyasaka, Hiroteru Hayashi, Daisuke Kubota, Shoutaro Arakawa, Yu Yamashita, Taiki Neyatani, Mitsuru Saito

**Affiliations:** ^1^ Department of Orthopaedic Surgery The Jikei University School of Medicine Tokyo Minato‐ku Japan

**Keywords:** Coronal Plane Alignment of the Knee, Larsen score, rheumatoid arthritis, side‐to‐side agreement, total knee arthroplasty

## Abstract

**Purpose:**

The Coronal Plane Alignment of the Knee classification estimates constitutional coronal alignment. In rheumatoid arthritis knees, disease‐related structural changes may affect the bony parameters used for this classification. This study described its distribution and side‐to‐side agreement in rheumatoid arthritis knees and explored radiographic differences associated with discordance.

**Methods:**

This single‐centre retrospective study included 57 patients and 93 knees treated with unilateral or bilateral total knee arthroplasty for rheumatoid arthritis between January 2014 and December 2024. Classification type was determined from preoperative standing full‐length anteroposterior radiographs. Side‐to‐side agreement was assessed in 36 bilaterally evaluable patients using complete agreement and unweighted Cohen's *κ*. Side‐to‐side differences in the medial proximal tibial angle (MPTA), lateral distal femoral angle (LDFA), joint line convergence angle (JLCA) and Larsen score were compared between agreement and discordance groups.

**Results:**

In 93 knees, Type I was most frequent at 36.6% (34 of 93), followed by Type II at 25.8% (24 of 93) and Type III at 24.7% (23 of 93). Types VII–IX were not observed. Complete side‐to‐side agreement was 36.1% (13 of 36), and unweighted Cohen's *κ* was 0.152 (95% confidence interval, −0.050 to 0.356; *p* = 0.093). Side‐to‐side differences in the MPTA, LDFA and JLCA did not differ significantly between groups. A Larsen score difference of at least 1 was more frequent in the discordance group than in the agreement group (78.3% [18 of 23] vs. 38.5% [5 of 13]; *p* = 0.030), with an odds ratio of 5.760 (95% confidence interval, 1.290–25.640).

**Conclusions:**

The classification showed limited side‐to‐side agreement in rheumatoid arthritis knees, and discordance was associated with asymmetric radiographic structural damage, indicating limited reliability as a standalone surrogate for constitutional alignment or contralateral knee morphology.

**Level of Evidence:**

Level IV.

**Abbreviations**
aHKA: arithmetic hip–knee–ankle angle
bDMARD: biological disease‐modifying antirheumatic drug
BHA: bipolar hemiarthroplasty
BMD: bone mineral density
CI: confidence interval
CPAK: Coronal Plane Alignment of the Knee
CRP: C‐reactive protein
csDMARD: conventional synthetic disease‐modifying antirheumatic drug
CT: computed tomography
ICC: intraclass correlation coefficient
JLCA: joint line convergence angle
JLO: joint line obliquity
LDFA: lateral distal femoral angle
MPTA: medial proximal tibial angle
OA: osteoarthritis
RA: rheumatoid arthritis
THA: total hip arthroplasty
TKA: total knee arthroplasty

## INTRODUCTION

The Coronal Plane Alignment of the Knee (CPAK) classification, proposed by MacDessi et al., is a framework for estimating constitutional knee alignment. It classifies coronal knee phenotypes into Types I–IX based on the arithmetic hip–knee–ankle angle (aHKA) and joint line obliquity (JLO), which are derived from the medial proximal tibial angle (MPTA) and lateral distal femoral angle (LDFA) measured on full‐length standing radiographs of the lower extremity [6, 16, 17].

CPAK distribution has been reported to vary according to country, region and study population [19, 25]. Even within the same population, distributional differences associated with patient characteristics, such as sex, have been reported [8, 21]. Limitations of interpreting the contralateral knee as a reference in surgical planning have also been described [1, 3, 28], and limited bilateral agreement has been reported for CPAK classification [23]. Regarding the clinical application of CPAK classification, the CPAK phenotype and its components, aHKA and JLO, have been reported to be insufficient for predicting preoperative tibiofemoral gaps [9]. In addition, bony alignment may affect patient‐specific laxity phenotypes [5]. Therefore, when CPAK classification is used as a bony coronal plane classification, individual soft‐tissue factors, such as gap and laxity, should also be considered.

Rheumatoid arthritis (RA) and osteoarthritis (OA) have traditionally been recognized as representative diseases that cause arthritis and joint deformity. However, in RA knees, the MPTA and LDFA that define CPAK classification may be affected by disease‐related changes, including synovitis‐related bone erosion, joint surface destruction, collapse and secondary degenerative changes [4]. Therefore, caution is required when interpreting CPAK classification obtained from RA knees as pre‐disease constitutional alignment. When interpreting CPAK classification in RA knees, it is necessary to consider not only MPTA and LDFA but also the joint line convergence angle (JLCA), which may complement intra‐articular and soft‐tissue factors under weight‐bearing conditions [15, 27], and radiographic structural damage indicators that can be evaluated at the knee level. However, most studies on CPAK classification have focused on OA knees, and reports on CPAK classification in RA knees remain limited [7].

This study aimed to describe the distribution of CPAK classification in patients who underwent unilateral or bilateral total knee arthroplasty (TKA) for RA and to evaluate side‐to‐side agreement of CPAK classification in bilaterally evaluable patients. In addition, to explore the background of side‐to‐side discordance in CPAK classification, we evaluated associations with side‐to‐side differences in MPTA, LDFA, JLCA and Larsen score, one of the radiographic indicators of joint destruction in RA.

## MATERIALS AND METHODS

### Study design and participants

This was a single‐centre retrospective observational study selecting patients who underwent unilateral or bilateral TKA for RA between January 2014 and December 2024. Eligibility was restricted a priori to Japanese patients because CPAK distribution has been reported to vary according to ethnicity [23]. Patients were assessed for eligibility, to exclude patients with previous arthroplasty or fracture. Patients with bilaterally evaluable radiographs were selected for side‐to‐side agreement analysis.

This study was approved by the institutional review board of our institution (Approval No. 32‐273 [12916]). Because of the retrospective design, the requirement for written informed consent was waived, and an opt‐out method was used to provide participants with the opportunity to decline participation.

### Radiographic measurements and Larsen grading

Preoperative standing full‐length anteroposterior radiographs of the lower extremity, obtained with the patella facing forward according to the principles described by Paley et al., were used for the analysis [20]. On each radiograph, the MPTA, LDFA and JLCA were measured to one decimal place using a digital measurement software (FUJIFILM OP‐A version 2.140; Fujifilm Corporation). MPTA was defined as the medial angle between the tibial mechanical axis and the tangent to the proximal tibial joint surface. LDFA was defined as the lateral angle between the femoral mechanical axis and the tangent to the distal femoral condyles. JLCA was defined as the angle between the tangent to the distal femoral condyles and the tangent to the proximal tibial joint surface. Radiographs in which the hip centre, knee centre, ankle centre, distal femoral joint line or proximal tibial joint line required for measurement could not be identified were excluded from the analysis. Larsen score was assessed on the same preoperative standing full‐length anteroposterior radiographs as a radiographic indicator of joint destruction in RA knees, using a six‐grade scale from 0 to 5 according to the Larsen grading system [11, 12].

### Definition of CPAK

The aHKA was defined as MPTA − LDFA, and JLO was defined as MPTA + LDFA. For aHKA, values from −2° to +2° were defined as neutral, values less than −2° as varus and values greater than +2° as valgus. For JLO, values from 177° to 183° were defined as neutral, values less than 177° as apex distal and values greater than 183° as apex proximal. CPAK Types I–IX were determined according to the combination of aHKA (varus/neutral/valgus) and JLO (apex distal/neutral/apex proximal) [6, 16, 17].

### Outcome measures

The primary outcomes were the distribution of CPAK classification. Side‐to‐side agreement was defined as both knees showing the same CPAK type and was evaluated using the complete agreement rate and unweighted Cohen's *κ* coefficient.

As secondary outcomes, continuous values of MPTA, LDFA, aHKA, JLO and JLCA were described. In bilaterally evaluable patients, the absolute side‐to‐side differences in MPTA and LDFA, which define CPAK classification, were calculated as ΔMPTA and ΔLDFA, respectively. In addition, side‐to‐side differences in JLCA and Larsen score were evaluated as supplementary indicators that are not components of CPAK classification. ΔJLCA was calculated as the absolute side‐to‐side difference in JLCA, and ΔLarsen was calculated as the absolute side‐to‐side difference in Larsen score. ΔLarsen was categorized as ΔLarsen = 0 or ΔLarsen ≥1.

To explore the background of side‐to‐side discordance in CPAK classification, ΔMPTA, ΔLDFA, ΔJLCA and the proportion of patients with ΔLarsen ≥1 were compared between the CPAK agreement and discordance groups. Clinical factors potentially associated with side‐to‐side discordance were also examined by comparing RA disease duration and the following variables assessed at the time of surgery between the CPAK agreement and discordance groups: C‐reactive protein (CRP) level; use of prednisolone, conventional synthetic disease‐modifying antirheumatic drugs (csDMARDs), biological disease‐modifying antirheumatic drugs (bDMARDs); and bone mineral density (BMD) at the lumbar spine and femoral neck, as assessed using dual‐energy X‐ray absorptiometry.

Furthermore, to evaluate whether JLCA and Larsen score, which are supplementary indicators and not components of CPAK classification, were associated with the background of side‐to‐side CPAK discordance, the associations of ΔJLCA with ΔMPTA and ΔLDFA were assessed, and ΔMPTA and ΔLDFA were compared between the ΔLarsen = 0 and ΔLarsen ≥1 groups. In addition, to assess the relationship between these supplementary indicators, ΔJLCA was compared between the ΔLarsen = 0 and ΔLarsen ≥1 groups.

### Statistical analysis

Descriptive statistics were used to summarize the CPAK distribution and radiographic parameters in all 93 knees. Continuous variables are presented as means ± standard deviations or medians [interquartile ranges], and categorical variables are presented as numbers and percentages.

Side‐to‐side agreement of CPAK classification in bilaterally evaluable patients was assessed using the complete agreement rate and unweighted Cohen's *κ* coefficient. The 95% confidence interval (CI) for the *κ* coefficient was calculated using the asymptotic standard error, and a two‐sided *p* value for *κ* = 0 was calculated.

Comparisons between the CPAK agreement and discordance groups and between the ΔLarsen = 0 and ΔLarsen ≥1 groups were performed using the Mann–Whitney *U* test for continuous and ordinal variables. Categorical variables were compared using Fisher's exact test. The association between CPAK discordance and ΔLarsen ≥1 was assessed using the odds ratio and 95% CI. The associations of ΔJLCA with ΔMPTA and ΔLDFA were evaluated using Spearman's rank correlation coefficient.

No correction for multiple comparisons was applied in the additional group comparisons or correlation analyses. This was a retrospective study using eligible cases within the study period, and no a priori sample size calculation was performed. A *p* value < 0.05 was considered statistically significant. Statistical analyses were performed using SPSS Statistics version 29.0.2.0 (IBM Japan, Ltd.).

### Measurement reliability

For interobserver reliability, six orthopaedic surgeons involved in knee care independently measured MPTA, LDFA and JLCA in 10 randomly selected knees under blinded conditions. Interobserver intraclass correlation coefficients (ICCs) were calculated using a two‐way random‐effects model for absolute agreement and are presented with 95% CIs [10]. For intraobserver reliability, the same evaluator repeated the measurements in 20 knees after an interval of 3 months, and intraobserver ICCs were calculated with 95% CIs.

For interobserver reliability of the Larsen score, two evaluators independently assessed the Larsen score in all 93 knees under blinded conditions, and weighted Cohen's *κ* coefficient was calculated.

## RESULTS

### Study population and radiographic parameters

Seventy‐eight patients underwent unilateral or bilateral TKA for RA. 156 knees were assessed for eligibility. Twenty‐one patients were excluded: one non‐Japanese patient, six patients with a history of bilateral total hip arthroplasty or bipolar hemiarthroplasty and 14 patients without eligible preoperative full‐length standing radiographs of the lower extremity. The final analysis included 57 patients and 93 knees (Figure 1). Patient characteristics and cohort composition are shown in Table 1. The mean age was 68.9 ± 10.2 years; 49 patients (86.0%) were female, eight patients (14.0%) were male, and the mean body mass index was 24.6 ± 4.1 kg/m^2^. Radiographic parameters are shown in Table 2. MPTA, LDFA, aHKA, JLO and JLCA were 86.0 ± 3.2°, 86.5 ± 2.7°, −0.5 ± 4.5°, 172.6 ± 3.8° and 1.7 ± 2.7°, respectively.

**Figure 1:**
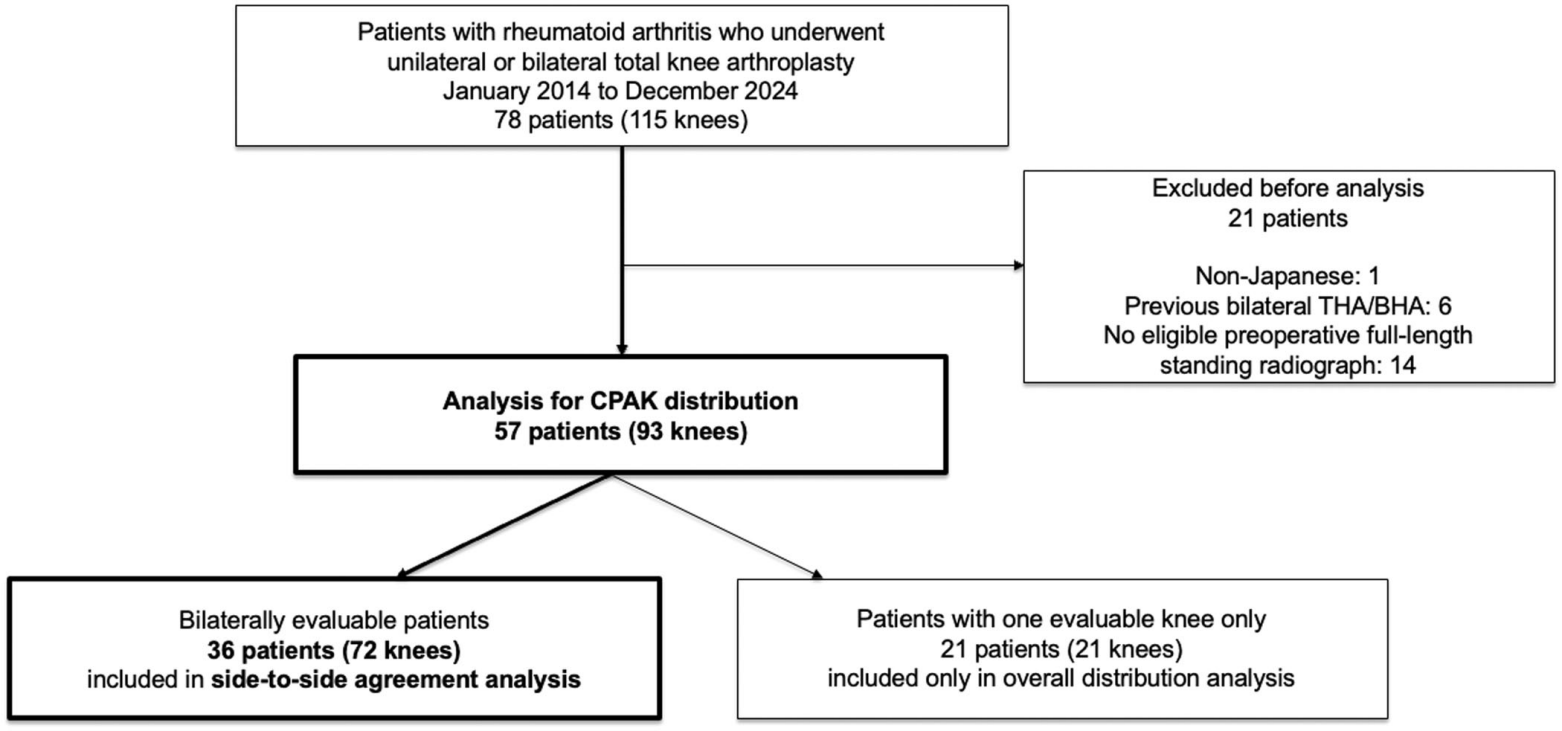
Patient selection flow diagram. BHA, bipolar hemiarthroplasty; THA, total hip arthroplasty.

**Table 1:** Patient characteristics and cohort composition.

Variable	Value
Patient‐level characteristics	
Patients, *n*	57
Age, years	68.9 ± 10.2
Female sex, *n* (%)	49 (86.0)
Male sex, *n* (%)	8 (14.0)
Body mass index, kg/m^2^	24.6 ± 4.1
Cohort composition	
Bilaterally evaluable patients, *n* (%)	36 (63.2)
Bilaterally evaluable knees, *n*	72
Patients with one evaluable knee only, *n* (%)	21 (36.8)
Knees from patients with one evaluable knee only, *n*	21
Knee‐level composition	
Knees, *n*	93
TKA knees, *n* (%)	70 (75.3)
Contralateral knees without TKA, *n* (%)	23 (24.7)

*Note*: Values are presented as mean ± standard deviation or number of cases (%), as appropriate. Percentages for patient‐level variables were calculated using 57 patients as the denominator, and percentages for knee‐level variables were calculated using 93 knees as the denominator. Bilaterally evaluable patients were included in side‐to‐side agreement analyses, whereas patients with one evaluable knee only were included only in the overall distribution analysis.

Abbreviation: TKA, total knee arthroplasty.

**Table 2:** Radiographic parameters in all knees.

Parameter	Value (*n* = 93)
MPTA, °	86.0 ± 3.2
LDFA, °	86.5 ± 2.7
aHKA, °	−0.5 ± 4.5
JLO, °	172.6 ± 3.8
JLCA, °	1.7 ± 2.7

*Note*: Values are presented as mean ± standard deviation. All parameters were measured on preoperative standing full‐length anteroposterior radiographs.

Abbreviations: aHKA, arithmetic hip–knee–ankle angle; JLCA, joint line convergence angle; JLO, joint line obliquity; LDFA, lateral distal femoral angle; MPTA, medial proximal tibial angle.

### CPAK distribution in all knees

The CPAK distribution in all 93 knees was as follows: Type I, 36.6% (*n* = 34); Type II, 25.8% (*n* = 24); Type III, 24.7% (*n* = 23); Type IV, 6.5% (*n* = 6); Type V, 3.2% (*n* = 3); and Type VI, 3.2% (*n* = 3) (Figure 2). Types I–III accounted for 87.1% (*n* = 81) of all knees. No knees were classified as Types VII–IX.

**Figure 2:**
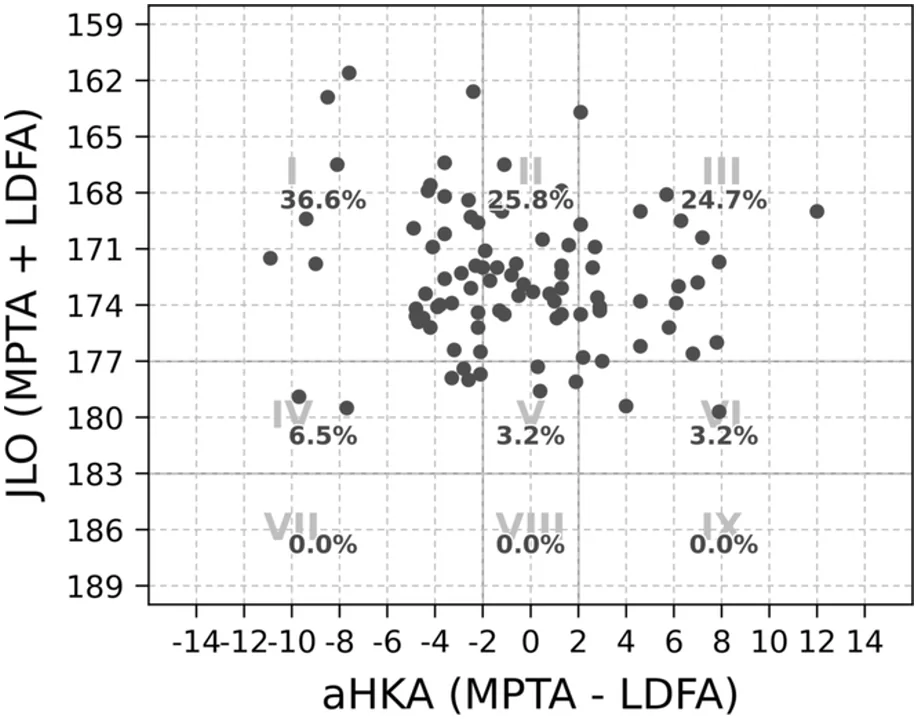
Coronal Plane Alignment of the Knee (CPAK) classification in rheumatoid arthritis knees. The horizontal axis represents the arithmetic hip–knee–ankle angle (aHKA), and the vertical axis represents joint line obliquity (JLO). Each point corresponds to one knee (*n* = 93). Percentages indicate the proportion of knees in each CPAK type. LDFA, lateral distal femoral angle; MPTA, medial proximal tibial angle.

### Side‐to‐side agreement of CPAK classification

A total of 36 patients (72 knees) were bilaterally evaluable, and complete side‐to‐side agreement of CPAK classification was observed in 36.1% (13 of 36), whereas discordance was observed in 63.9% (23 of 36). The unweighted Cohen's *κ* coefficient was 0.152 (95% CI, −0.050 to 0.356; *p* = 0.093). The cross‐tabulation of right and left CPAK classifications is shown in Table 3.

**Table 3:** Side‐to‐side agreement of CPAK classification in bilaterally evaluable patients.

(a) Summary	
Measure	Value
Complete side‐to‐side agreement	36.1% (13/36)
Discordance	63.9% (23/36)
Unweighted Cohen's *κ*	0.152
95% confidence interval	−0.050 to 0.356
*p* value	0.093

(b) Cross‐tabulation						
Right knee\Left knee	I	II	III	IV	V	VI
I	5	5	0	1	1	1
II	5	1	3	1	1	0
III	1	2	6	0	0	0
IV	0	0	0	1	0	0
V	0	0	0	1	0	0
VI	0	0	0	1	0	0

*Note*: Values are presented as the number of patients. Rows and columns indicate CPAK types of the right and left knees, respectively. Complete agreement was defined as both knees being classified as the same CPAK type. The *p* value for unweighted Cohen's *κ* was calculated against a null hypothesis of *κ* = 0.

Abbreviation: CPAK, Coronal Plane Alignment of the Knee.

### Comparison of side‐to‐side differences according to CPAK agreement

Comparisons of side‐to‐side differences between the CPAK agreement group (*n* = 13) and discordance group (*n* = 23) are summarized in Table 4. ΔMPTA was 1.8° [1.3°, 2.7°] and 2.4° [1.5°, 5.3°], ΔLDFA was 2.3° [1.9°, 3.5°] and 1.2° [0.9°, 2.7°], and ΔJLCA was 1.2° [0.5°, 2.0°] and 1.5° [0.9°, 3.1°] in the agreement and discordance groups, respectively, with no significant differences. In contrast, ΔLarsen ≥1 was observed in 38.5% (5 of 13) of the agreement group and 78.3% (18 of 23) of the discordance group and was significantly more frequent in the discordance group (*p* = 0.030). The odds ratio for ΔLarsen ≥1 in the discordance group was 5.760 (95% CI, 1.290–25.640). In exploratory comparisons of clinical factors, no significant differences were observed between the CPAK agreement and discordance groups in RA disease duration or in any of the following variables assessed at the time of surgery: CRP level; use of prednisolone, csDMARDs, bDMARDs; and BMD at the lumbar spine and femoral neck (Table S1).

**Table 4:** Comparison of side‐to‐side differences according to CPAK agreement.

Variable	Agreement group (*n* = 13)	Discordance group (*n* = 23)	*p* value
ΔMPTA, °	1.8 [1.3, 2.7]	2.4 [1.5, 5.3]	0.267
ΔLDFA, °	2.3 [1.9, 3.5]	1.2 [0.9, 2.7]	0.123
ΔJLCA, °	1.2 [0.5, 2.0]	1.5 [0.9, 3.1]	0.267
ΔLarsen ≥1, *n* (%)	5 (38.5)	18 (78.3)	0.030

*Note*: Values are presented as median [interquartile range] or number of cases (%), as appropriate. Δ indicates the absolute side‐to‐side difference between the right and left knees. *p* values were calculated using the Mann–Whitney *U* test for continuous variables and Fisher's exact test for categorical variables. The odds ratio for CPAK discordance associated with ΔLarsen ≥1 was 5.760 (95% confidence interval, 1.290–25.640).

Abbreviations: CPAK, Coronal Plane Alignment of the Knee; JLCA, joint line convergence angle; LDFA, lateral distal femoral angle; MPTA, medial proximal tibial angle.

Furthermore, ΔJLCA was not significantly correlated with ΔMPTA or ΔLDFA (*ρ* = 0.196, *p* = 0.252; *ρ* = 0.157, *p* = 0.362). In comparisons between the ΔLarsen = 0 and ΔLarsen ≥1 groups, ΔMPTA tended to be larger in the ΔLarsen ≥1 group, but the difference did not reach statistical significance (*p* = 0.055). No significant differences were observed in ΔLDFA or ΔJLCA.

### Measurement reliability

Measurement reliability for MPTA, LDFA and JLCA is shown in Data S1. The interobserver ICCs were 0.932 for MPTA, 0.941 for LDFA, and 0.982 for JLCA. The intraobserver ICCs were 0.964 for MPTA, 0.917 for LDFA and 0.989 for JLCA. For the interobserver reliability of the Larsen score, the weighted Cohen's *κ* coefficient was 0.751 (95% CI, 0.662–0.841).

## DISCUSSION

The main findings of this study were that CPAK classification in RA knees was concentrated in Types I–III, that complete side‐to‐side agreement was limited to 36.1% among bilaterally evaluable patients, and that ΔLarsen ≥1 was significantly more frequent in the CPAK‐discordant group, whereas ΔMPTA, ΔLDFA and ΔJLCA did not differ significantly between the CPAK agreement and discordance groups. These findings suggest that, in RA knees, CPAK classification has limited reliability as a standalone surrogate for pre‐disease constitutional alignment or the contralateral knee. In the following sections, we discuss the distribution of CPAK classification, side‐to‐side agreement, the relationship with disease‐related structural changes and limitations in interpreting CPAK classification in RA knees undergoing TKA.

In Asian TKA cohorts, Types I and II have been reported to be common, whereas the proportion of Type III has been relatively low [23]. In contrast, reports on CPAK classification in RA knees remain limited. Hirotomi et al. reported that, among 69 RA knees, Type I accounted for 36.2%, Type II for 26.1% and Type III for 16.0% [7]. In the present study, Type I was the most frequent type, accounting for 36.6% (34 of 93), followed by Type II at 25.8% (24 of 93) and Type III at 24.7% (23 of 93). The proportions of Types I and II were broadly similar to those reported by Hirotomi et al., whereas the proportion of Type III was somewhat higher in the present cohort.

RA knees have been reported to show diverse coronal plane deformities that differ from those observed in OA knees [18]. In addition, studies of knees undergoing TKA in patients with RA have reported changes in destructive patterns, suggesting that RA knees may include not only typical inflammatory joint destruction but also secondary OA‐like changes [4, 26]. On this basis, the CPAK distribution in RA knees may not solely reflect pre‐disease constitutional alignment, but may also reflect knee morphology affected by disease‐related changes.

Given this background, the present study evaluated not only CPAK distribution but also side‐to‐side agreement in bilaterally evaluable patients. Recently, limitations of using the contralateral knee as a reference in preoperative planning for TKA have been reported. Pujol et al. reported that CPAK agreement between the operative and contralateral knees was 38% among 76 patients scheduled for unilateral TKA [22]. Rahman et al. reported bilateral agreement of CPAK classification in 45% of 38 Asian patients who underwent TKA [23]. In contrast, previous reports on CPAK classification in RA knees have not sufficiently evaluated side‐to‐side agreement [7]. In the present study, complete side‐to‐side agreement of CPAK classification was limited to 36.1% (13 of 36) among 36 bilaterally evaluable patients with RA, and the unweighted Cohen's *κ* coefficient was 0.152. Cohen's *κ* coefficient is a chance‐corrected measure of agreement; therefore, the low value observed in this study indicates only slight agreement. These findings indicate that side‐to‐side agreement of CPAK classification is also limited in RA knees, showing a trend similar to previous reports in OA or TKA cohorts.

To examine radiographic factors associated with side‐to‐side discordance in CPAK classification, this study evaluated side‐to‐side differences in MPTA, LDFA, JLCA and Larsen score. JLCA is an indicator that may reflect intra‐articular deformity and soft‐tissue factors under weight‐bearing conditions, and associations with OA severity and intra‐articular deformity have been reported [15, 27]. However, in the present study, ΔJLCA did not differ significantly between the CPAK agreement and discordance groups, and ΔJLCA showed no clear association with ΔMPTA or ΔLDFA. This finding indicates that side‐to‐side discordance in CPAK classification in RA knees cannot be explained solely by intra‐articular or soft‐tissue factors represented by JLCA.

In contrast, ΔLarsen ≥1 was significantly more frequent in the CPAK‐discordant group. Larsen score is a radiographic indicator of structural joint destruction in RA [11, 12], and this finding indicates an association between side‐to‐side asymmetry in Larsen score and discordance in CPAK classification. Exploratory comparisons of clinical factors showed no significant differences between the CPAK agreement and discordance groups in RA disease duration, CRP level, medication use or BMD. However, the association between CPAK discordance and side‐to‐side differences in Larsen score should be considered exploratory and interpreted cautiously because the analysis was based on only 36 bilaterally evaluable patients, no correction for multiple comparisons was applied, the 95% CI for the odds ratio was wide, and no multivariable adjustment for confounding was performed. In addition, although ΔMPTA tended to be larger in the ΔLarsen ≥1 group, the difference did not reach statistical significance, and no clear difference was observed in ΔLDFA. Therefore, it could not be concluded that side‐to‐side differences in Larsen score were associated with CPAK discordance through either MPTA or LDFA.

These findings indicate limitations in the interpretation of CPAK classification in RA knees. CPAK classification is based on aHKA and JLO; however, even within the same CPAK type, the combination of MPTA and LDFA may differ. In addition, because CPAK classification applies categorical thresholds to continuous parameters derived from MPTA and LDFA, small side‐to‐side differences near the classification cut‐offs may result in different CPAK types even when the underlying coronal anatomy is similar. It has also been reported that CPAK classification alone cannot adequately identify segmental coronal deformity on the femoral or tibial side [14]. In the present study, although no clear group differences were observed in ΔMPTA or ΔLDFA and ΔLarsen ≥1 was significantly more frequent in the CPAK‐discordant group, no specific angular component associated with side‐to‐side CPAK discordance could be identified. Therefore, side‐to‐side discordance in CPAK classification in RA knees may have a complex background involving asymmetric structural destruction, rather than being attributable to a single angular component. Taken together, these findings indicate that, in RA knees, CPAK classification has limited reliability as a standalone surrogate for pre‐disease constitutional alignment or contralateral knee morphology.

Clinically, considering the present findings together with previous reports [5, 9] showing the limited ability of CPAK classification to predict tibiofemoral gaps and the importance of patient‐specific laxity phenotypes, CPAK classification should be interpreted together with knee‐specific radiographic morphology, the degree of structural joint damage, JLCA and soft‐tissue balance in preoperative planning and surgical decision‐making for TKA. Furthermore, there are limitations to regarding CPAK type as a standalone basis for estimating constitutional alignment or for coronal alignment and bone‐resection planning.

Recent studies have evaluated side‐to‐side coronal phenotype symmetry using functional knee phenotype classification [24], compared functional knee phenotypes with CPAK classification using three‐dimensional computed tomography (CT) reconstruction models [13] and examined the relationship between CPAK classification and alignment parameters in other planes or three‐dimensional knee morphology [2, 29]. These alternative phenotype‐based alignment classifications and three‐dimensional imaging assessments may provide information that is not fully captured by two‐dimensional CPAK classification, including side‐to‐side phenotype symmetry and three‐dimensional knee morphology involving sagittal and rotational planes. However, because these methods were not evaluated in the present study and were not directly compared with CPAK classification, their relative usefulness in RA knees requires further investigation.

This study has several limitations. First, this was a single‐centre retrospective cross‐sectional observational study with a limited sample size of 57 patients, and the side‐to‐side agreement analysis was limited to 36 bilaterally evaluable patients. Therefore, the generalizability of the present findings is limited. In addition, because of the observational nature of this study, the association between ΔLarsen and side‐to‐side discordance in CPAK classification should not be interpreted as evidence of a causal relationship.

Moreover, the analyses of clinical factors were exploratory comparisons conducted in the 36 bilaterally evaluable patients, and because no multivariable adjustment for confounding was performed, residual confounding cannot be excluded. Second, the study population was limited to patients who underwent at least unilateral TKA for RA. Third, although selected clinical factors, including disease duration, medication use and BMD, were examined in exploratory analyses, systemic RA activity, such as Disease Activity Score 28, and detailed longitudinal treatment‐related factors were not systematically evaluated in all patients. Fourth, this study was based on two‐dimensional assessment using standing full‐length anteroposterior radiographs of the lower extremity. Alternative phenotype‐based alignment classifications were not evaluated or directly compared with CPAK classification. In addition, because three‐dimensional imaging assessment was not performed, sagittal and rotational morphology and the influence of three‐dimensional bone destruction could not be evaluated. Therefore, there are limitations to interpreting the side‐to‐side discordance in CPAK classification observed in this study in relation to three‐dimensional constitutional alignment. Finally, tibiofemoral gaps and patient‐specific soft‐tissue laxity were not directly evaluated in this study.

## CONCLUSION

Complete side‐to‐side agreement of CPAK classification was limited in RA knees, with an observed association between CPAK discordance and asymmetry in radiographic structural damage. These findings suggest that CPAK classification has limited reliability as a standalone surrogate for pre‐disease constitutional alignment or contralateral knee morphology in RA knees.

## AUTHOR CONTRIBUTIONS

Toshiyuki Omori contributed to the study design, data collection, data analysis and drafting of the manuscript as the principal investigator. Tomohiro Kayama contributed to the data collection, data analysis and English language editing. Teruyuki Miyasaka, Hiroteru Hayashi, Daisuke Kubota, Shoutaro Arakawa, Yu Yamashita and Taiki Neyatani contributed to the data collection and data analysis. Mitsuru Saito provided overall supervision of the study as well as critical revision of the manuscript. All authors read and approved the final manuscript.

## FUNDING INFORMATION

The authors have no funding to report.

## CONFLICT OF INTEREST STATEMENT

The authors declare no conflicts of interest. However, the Department of Orthopaedic Surgery at The Jikei University School of Medicine receives scholarship donations from Zimmer Biomet, Kyocera Corporation and Smith & Nephew.

## ETHICS STATEMENT

All procedures performed in studies involving human participants were in accordance with the ethical standards of the institutional research committee and with the 1964 Helsinki Declaration and its later amendments or comparable ethical standards. This study was approved by the Institutional Review Board of The Jikei University School of Medicine (Approval No. 32‐273(12916)). The requirement for written informed consent was waived by the Institutional Review Board of The Jikei University School of Medicine based on the retrospective nature of the study; an opt‐out method was used to provide information to the participants.

## Supporting information

Supplementary Data 1.

Supplementary Table 1.

## Data Availability

The data that support the findings of this study are available from the corresponding author upon reasonable request.
