# Supplementary material for: CPAK classification shows limited side‐to‐side agreement and limited reliability as a standalone surrogate for constitutional alignment in rheumatoid arthritis knees
Source: J Exp Orthop. 2026 Oct 6;13(4):e70942. doi: 10.1002/jeo2.70942 (PMC13641184; doi:10.1002/jeo2.70942)
Supplement: Supplementary file 1 [file JEO2-13-e70942-s001.docx]

**Supplementary data.**

**Measurement reliability**

| **Parameter** | **Interobserver ICC** | **95% confidence interval** | **Intraobserver ICC** | **95% confidence interval** |
| --- | --- | --- | --- | --- |
| MPTA | 0.932 | 0.850–0.980 | 0.964 | 0.913–0.985 |
| LDFA | 0.941 | 0.868–0.982 | 0.917 | 0.805–0.966 |
| JLCA | 0.982 | 0.958–0.995 | 0.989 | 0.972–0.995 |

| **Parameter** | **Weighted Cohen’s kappa** | **95% confidence interval** |
| --- | --- | --- |
| Larsen score | 0.751 | 0.662–0.841 |

Interobserver reliability was assessed in 10 randomly selected knees by six independent orthopaedic surgeons using a two-way random-effects model for absolute agreement.

Intraobserver reliability was assessed in 20 knees by repeated measurements by the same observer after a 3-month interval.

Reliability for MPTA, LDFA, and JLCA is presented as intraclass correlation coefficients.

Reliability for Larsen score was assessed in all 93 knees using weighted Cohen’s kappa.

Abbreviations: ICC, intraclass correlation coefficient; JLCA, joint line convergence angle; LDFA, lateral distal femoral angle; MPTA, medial proximal tibial angle.
