# Supplementary material for: CPAK classification shows limited side‐to‐side agreement and limited reliability as a standalone surrogate for constitutional alignment in rheumatoid arthritis knees
Source: J Exp Orthop. 2026 Oct 6;13(4):e70942. doi: 10.1002/jeo2.70942 (PMC13641184; doi:10.1002/jeo2.70942)
Supplement: Supplementary file 2 [file JEO2-13-e70942-s002.docx]

**Supplementary Table 1.** Exploratory comparison of clinical factors between the CPAK agreement and discordance groups

| **Variable** | **CPAK agreement group (n = 13)** | **CPAK discordance group (n = 23)** | ***p* value** |
| --- | --- | --- | --- |
| RA disease duration, years | 6 [1, 22] | 7 [2, 21] | 0.869 |
| CRP, mg/dL | 0.60 [0.16, 1.51] | 0.35 [0.08, 1.26] | 0.767 |
| Prednisolone use, n (%) | 5 (38.5) | 9 (39.1) | 1.000 |
| csDMARD use, n (%) | 8 (61.5) | 17 (73.9) | 0.475 |
| bDMARD use, n (%) | 7 (53.8) | 10 (43.5) | 0.730 |
| Lumbar spine BMD, g/cm² | 1.124 [1.004, 1.210] | 1.111 [0.928, 1.261] | 0.961 |
| Femoral neck BMD, g/cm² | 0.818 [0.683, 0.998] | 0.733 [0.672, 0.917] | 0.576 |

Data are presented as median [interquartile range] or number (%). Continuous variables were compared using the Mann–Whitney U test, and categorical variables were compared using Fisher’s exact test.

Abbreviations: CPAK, coronal plane alignment of the knee; RA, rheumatoid arthritis; CRP, C-reactive protein; csDMARD, conventional synthetic disease-modifying antirheumatic drug; bDMARD, biological disease-modifying antirheumatic drug; BMD, bone mineral density.
